# Comparison of Different Waves during the COVID-19 Pandemic: Retrospective Descriptive Study in Thailand

**DOI:** 10.1155/2021/5807056

**Published:** 2021-10-08

**Authors:** Jadsada Kunno, Busaba Supawattanabodee, Chavanant Sumanasrethakul, Budsaba Wiriyasivaj, Sathit Kuratong, Chuthamat Kaewchandee

**Affiliations:** ^1^Department of Research and Medical Innovation, Faculty of Medicine Vajira Hospital, Navamindradhiraj University, Bangkok, Thailand; ^2^Department of Urban Medicine, Faculty of Medicine Vajira Hospital, Navamindradhiraj University, Bangkok, Thailand; ^3^Department of Obstetrics and Gynecology, Faculty of Medicine Vajira Hospital, Navamindradhiraj University, Bangkok, Thailand; ^4^Department Medicine, Faculty of Medicine Vajira Hospital, Navamindradhiraj University, Bangkok, Thailand

## Abstract

**Background:**

Coronavirus disease (COVID-19) is an infectious disease caused by a newly discovered coronavirus. An outbreak is called an epidemic when there is a sudden increase in cases. Many countries have experienced a two-wave pattern in the reported cases of COVID-19. The spread of COVID-19 in Thailand was a cluster event distributed over multiple locations. This study aims to compare the characteristics of different waves during the COVID-19 pandemic in Thailand.

**Methods:**

A retrospective cohort study was conducted from January 2020 to May 2021 (17 months) to determine the number of COVID-19 screenings and confirmed cases and deaths as well as sociodemographic characteristics such as gender, age, nationality, and source population at risk factors. The categorical data were compared using a chi-square test.

**Results:**

Three waves of the COVID-19 pandemic occurred within 17 months in Thailand, and the number of cases increased by over 100,000 due to source population at risk factors such as close contact with a previously confirmed patient, community risk, cluster communities, and active and community surveillance. The chi-square test revealed significant differences between the three waves (*p* < 0.01).

**Conclusion:**

Significant differences between pandemic phases or waves may be due to weak social distancing policies and the lack of public health interventions. A COVID-19 vaccination plan is needed for people at risk of suffering severe symptoms and the general population in outbreak areas to increase immunity.

## 1. Introduction

The initial cases of the novel coronavirus (2019-nCoV) occurred in Wuhan, Hubei Province, China, in December 2019 and January 2020 [1]. In December 2019, a cluster of patients with pneumonia of unknown cause was linked to a seafood wholesale market in Wuhan, China [1, 2]. Globally, there have been more than 177 million cases, and the number likely to decline is below 500,000 people per day [3,  4]. Many outbreaks are still occurring in India and South America [3, 4]. The total number of deaths exceeds 3.8 million people; however, daily deaths have decreased to below 10,000 people per day [3]. Most people infected with the COVID-19 virus had experienced mild-to-moderate respiratory illness and recovered without special treatment [5]. Older people with underlying medical problems such as cardiovascular disease, diabetes, chronic respiratory disease, and cancer are more likely to develop serious illnesses [5]. The COVID-19 virus spreads primarily through droplets of saliva or discharge from the nose when an infected person coughs or sneezes, so practicing respiratory etiquette is important [6].

Many countries have experienced multiple waves of coronavirus outbreaks. During the 2020 pandemic, empirical data show that characteristics varied between waves [7]. In comparison with the second wave, the proportion of local clusters (24.8% vs 45.7%) was lower in the third wave, and personal contact transmission (38.5% vs 25.9%) and unknown routes of transmission (23.5% vs 20.8%) were higher [8]. Consequently, many governments and health authorities, including the WHO, have been actively educating people to take preventive measures to reduce the spread of the virus, including lockdown measures [9, 10].

Waves in Thailand [11, 12] have been traced to superspreading events at entertainment establishments, pubs, bars, Karaoke lounges, and various types of gambling venues in different country regions. These events led to an expansion of the spread of COVID-19 to many provinces since the risk locations were sites that attracted crowding, extended interactions, and high turnover. An outbreak in March 2020 was associated with attendees at a boxing stadium in Bangkok who spread the virus to other provinces as they travelled home or on business. One of the factors behind the outbreak was the dense living conditions of the migrants in the surrounding community and the lack of personal precautions to prevent spread.

This study compared the characteristics of different waves during the COVID-19 pandemic in Thailand, where more than 150,000 COVID-19 cases have been confirmed. Although the daily number of infections in the community has stabilised at around 2,000 per day, locally transmitted cases have been confirmed in most provinces. In Thailand, the spread of COVID-19 was a cluster event distributed over multiple locations. This event had the effect of distributing COVID-19 to a large number of provinces [11]. The number of deaths during the current wave has reached 1,031 cases for a mortality rate of 0.82% [12]. During the current wave, outbreaks have been reported in closed areas with crowds of people such as establishments, prisons, markets, shopping malls, and communities [7].

## 2. Methods

### 2.1. Study Design

We conducted a retrospective cohort study of all test screenings and cases of COVID-19 in Thailand. Information was recorded between January 2020 and May 2021 (17 months) [11, 12]. All records were fully anonymised before the researchers accessed them. The ethics committee of Faculty of Medicine Vajira Hospital, Navamindradhiraj University, Bangkok, Thailand, approved this study (COE: 011/2021X).

### 2.2. Data Collection

COVID-19 data gathered by the Department of Disease Control, Ministry of Public Health, Thailand, between January 2020 and May 2021 were collected, including time period, number of test screenings, number of confirmed cases and deaths, and sociodemographic characteristics, including gender, age, nationality, and sources population of risk factors.

The study period was stratified based on the month of test screening and diagnosis to identify temporal trends in cases. Population data were divided into five phases:

  Phase I: January–February 2020
  Phase II: March–May 2020 (first wave)
  Phase III: June–November 2020
  Phase IV: December 2020–March 2021 (second wave)
  Phase V: April–May 2021 (third wave).

### 2.3. Study Population

The COVID-19 infection database was queried to identify all recorded ages, gender, nationality, and at-risk source populations. Cases in which such information was missing were excluded.

We stratified the population based on age (infants [0-1 year], toddlers [1–3 years], preschoolers [4-5  years], middle childhood [6–12 years], adolescents [13–15 years], and adults: 16–18 years, 19–24 years, 25–30 years, 31–35 years, 36–40 years, 41–50 years, 51–60 years, and >60 years) and gender (males and females). We stratified nationality into Thai, migrant worker (Myanmar, Khmer, and Laotian), and foreigner categories.

**Figure 1:**
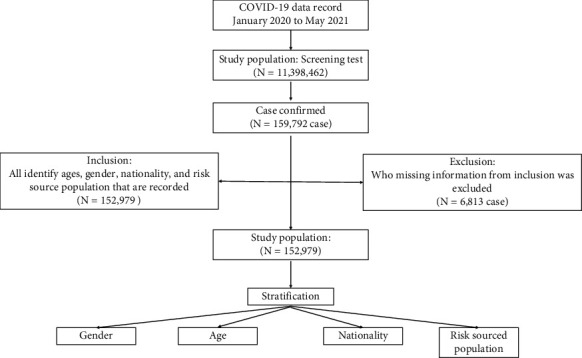
Derivation of the study population.

The at-risk source population of risk factors was stratified based on close contact with a previously confirmed patient, status as a health care worker, community risk (such as enclosed space), cluster community, active and community surveillance, and state quarantine (Figure 1).

### 2.4. Statistical Analysis

We summarised the characteristics for continuous and categorical data as numbers and percentages. Characteristics were compared using descriptive statistics, and categorical data were compared using a chi-square test, whereby *p* < 0.05 was considered to indicate statistical significance. Statistical analysis was performed using the Statistical Package for the Social Sciences Program (SPSS), version 22.

## 3. Results

### 3.1. Cases and Deaths during the COVID-19 Pandemic

From January 2020 to May 2021, approximately 11 million people were screened for COVID-19 in Thailand, and there were approximately 150,000 confirmed cases and 1,000 deaths within the 17-month period (Table 1).

During Phase I (January–February 2020), approximately 3.5 million screening tests confirmed fewer than 100 cases, and there were no deaths. During the first wave in Phase II (March–May 2020), the number of new cases increased to approximately 3,040 cases, and there were nearly 60 recorded deaths. Phase III (June–November 2020) was characterised by low levels of new cases (approximately 938 cases) and deaths (3 cases). However, a second wave occurred during Phase IV (December 2020–March 2021), when the number of new cases increased to more than 20,000 cases, approximately 30 recorded deaths. A third wave—which continues into the present—can be observed during Phase V (April–May 2021), when the number of new cases increased to more than 100,000, and 1,000 deaths were documented (Table 2).

### 3.2. Demographics

Demographic data of the study population are shown in Table 2. Approximately 51.5% and 48.5% of individuals were male and female, respectively. Ages ranged from one month to over 60 years. Nearly 21% of cases occurred among adults 25–30 years of age. Thai (77.4%) was the main nationality, distantly followed by migrant workers (Myanmar, Khmer, and Laotian) (21.1%), and other foreigners (1.5%). Data of the risk source populations revealed that most tested individuals had been in close contact with a previously confirmed patient (43.9%). In addition, other risk source populations were cluster communities (25.7%) and community risk (8.5%), active and community surveillance (19.9%), state quarantine (1.5%), and health care workers (0.4%).

### 3.3. Different Waves during the COVID-19 Pandemic

Table 2 illustrates demographic and case data across the five phases. The 3,040 cases during the first wave (Phase II) involved slightly more males (54.8%) than females (45.2%), and most of the ill were aged 25–30 (18.2%) and 41–50 (18.3%) years. Most patients were Thai (89.9%), and very few were foreigners (6.8%) or migrant workers (3.3%). The source population at risk factors assessment indicates that most patients reported close contact with a previously confirmed patient (52.6%), followed by community risk (37.7%).

The 22,000 cases during the second wave (Phase IV) comprised slightly more females (58.0%) than males (42.0%), and most patients were aged 25–30 (24.2%) and 31–35 (18.2%) years. In contrast to the first wave, most cases occurred among migrant workers (61.2%), followed by Thais (36.1%) and other foreigners (2.8%). The source population at risk factors assessment found that most cases arose from cluster communities (80%), followed by close contact with a previously confirmed patient (12.6%).

The 127,000 cases during the third wave (Phase V) comprised slightly more males (51.5%) than females (47.0%), and most patients were aged 25–30 (21.0%) and 41–50 (15.4%) years. Most patients were Thai (84.3%), followed by migrant workers (14.8%) and other foreigners (0.9%). The source population at risk factors assessment found that most cases resulted from close contact with a previously confirmed patient (49.2%), followed by active and community surveillance (23.7%).

**Figure 2:**
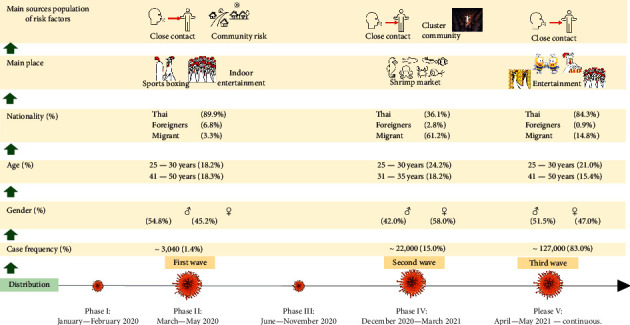
Summary for different waves during the COVID-19 pandemic in Thailand.

The proportion of confirmed cases in the different epidemic waves was 1.4% in the first wave, 15.0% in the second wave, and 83% in the third wave (Table 1). Thus, the number of cases was increased by more than 120,000 from the first to the third wave of the COVID-19 pandemic, and source population at risk factors assessments attributed cases to close contact with a previously confirmed patient, community risk, cluster community, and active and community surveillance (see Figure 2). The chi-square test found significant differences between the phases and waves (*p* < 0.01).

## 4. Discussion

This study conducted a retrospective cohort study of all test screenings and cases of the COVID-19 pandemic in Thailand from January 2020 to May 2021. The results show over 152,000 confirmed cases of COVID-19 and more than 1,000 deaths during that 17-month period. The COVID-19 pandemic has resulted in both deaths and recoveries; there is no room for mistakes, as one wrong or delayed decision can worsen the situation [13]. Higher testing rates have led to the identification of more cases; thus, differences in the identified number of cases and the actual number of cases largely depend on the extent of testing and diagnosis [14]. However, some studies suggest that increases in confirmed cases and deaths due to the coronavirus are associated with significantly higher market illiquidity and volatility and declining sentiment. The implementation of restrictions and lockdowns contributes to the deterioration of market liquidity and stability [15].

In the first wave (March–May 2020), most reported cases occurred among people of Thai nationality. In addition, this wave presented strain A.6; it was originally strain in Thailand. Severe illness was continuous cough, exhausted, tasteless tongue, difficulty breathing, do not smell, and fever greater than 37°C [16]. The main risk sources were close contact with a previously confirmed patient and community risk. From that time, the number of cases of COVID-19 in Thailand increased slowly but steadily due to both imported cases and local transmission. Since the first wave, clusters of outbreaks have been traced to superspreading events in sports venues or indoor entertainment establishments [12, 17].

The origin of the second wave (December 2020–March 2021) has been traced to a wholesale shrimp market. There was an increase in new cases during this period compared with the first wave. Most confirmed cases occurred among migrant labourers working in the market locality. Additionally, strain B.1.36.16 was presented; it was expected that this strain endemic to Thailand to replace the A.6 strain [16]. The main risk sources were cluster communities and close contact with a previously confirmed patient. One of the factors behind the outbreak was the dense living conditions of the migrants residing in the surrounding community and the lack of personal precautions to prevent infection spread [17]. Since then, the Thai government has implemented policies to support migrant workers living in Thailand. Migrant labourers have often been stigmatized and unjustly blamed for the spread of disease; however, in reality, they were one of the worst affected groups [18]. Early and timely interventions along with strengthened social distancing policies should be implemented to effectively suppress and control the COVID-19 pandemic [11].

During the third wave (April–May 2021 to present), superspreading events were identified at entertainment establishments, pubs, bars, karaoke lounges, and various types of gambling venues in different country regions. The number of cases increased to more than the first and second waves combined. The predominant nationality was Thai, followed by migrant workers. Additionally, strain Alpha-B.1.1.7 was presented; it can be spread much faster than other variants, severe illness and death may potentially cause more people to get sicker and to die, both strains Beta-B.1.351 (severe illness and death; current data do not indicate more severe illness or death than other variants) and Data-B.1.617.2 (severe illness and death, may cause more severe cases than the other variants) were found during third wave to present [16]. The main risk sources were close contact with a previously confirmed patient, active and community surveillance, and cluster communities. Due to the highly transmissible nature of COVID-19, delayed intervention may have led to rapid spread.

Hence, this study found significant differences between the three waves of the COVID-19 pandemic in Thailand. Our results indicate that the third wave is more serious than previous waves, which may be due to a lack of strong social distancing policies and public health interventions. Our findings differ from other studies showing that the first wave of COVID-19 pandemic had the most negative impact on public health. In contrast, the second wave evinced more stable evolutionary dynamics [19]. In addition, sufficient epidemiologic investigations and contact tracing could not be performed during the third wave, and there was a marked increase in the proportion of unknown routes of transmission [11, 20]. The reason for the clear differences across phases and waves is not yet known, although it has been suggested that a new variant of COVID-19 emerged in Thailand in the middle of 2021, and transmission to the general population was replicated across the country. A quantile regression model suggests that globalisation, settlement, and population characteristics related to high human mobility and interaction predict disease diffusion [21]. However, in this study, the most striking difference between the first, second, and third waves in Thailand was implementing public health interventions.

In terms of gender and age, they were significantly associated with differences across phases and waves (*p* < 0.001) of the COVID-19 pandemic in Thailand, but some studies presented that the effect of gender and age in perceived vulnerability to infectious diseases variables suggest that gender plays a role in young people only, where women presented higher perceived infectability and germ aversion than men [22]. However, the harm to children can occur in multiple, often hidden, ways, and exclusively focusing on the health effects of the infection misses the broader impact of the pandemic on children's lives [23]. In addition, differences in age range and severity of the disease have been reported—severe cases have affected more patients younger than 3 years and older than 60 years compared with previous waves. However, the comparative characteristics of the two waves remain largely unknown [12, 17]. Our study considered nationality as an independent risk factor. This study found that nationality was significantly associated with differences across phases and waves (*p* < 0.001). However, a systematic review and meta-analysis could not confirm ethnicity as an independent risk factor for negative outcomes in COVID-19 patients [24]. It is time to learn from the lessons of past disease outbreaks. Given the low-to-high-quality evidence indicating that ethnicity is not an independent risk factor, COVID-19 risk assessments should only consider ethnicity in conjunction with other risk factors, such as age or comorbidities. Hence, the COVID-19 pandemic deeply impacted all sociodemographic groups living in Thailand, including Thai, migrant workers, and foreigners across genders and all stages of life. The most prominent risk factors were close contact with a previously confirmed patient and community risk.

In fact, that source population at risk factor was significantly associated with differences across phases and waves (*p* < 0.001). The most prominent risk factors were close contact with a previously confirmed patient and community risk. The best way to prevent and slow transmission is to be well informed about the COVID-19 virus, the disease it causes, and how it spreads [9]. One study suggested that population density and population characteristics such as total population, older populations, and household size are strong predictors in early weeks but have a muted impact on reported COVID-19 diffusion over time [21]. Thus, social distancing would not be effective; screening and surveillance were expanded to try to detect potential outbreaks before they could ignite [17]. Protective measures include hand washing or using an alcohol-based rub frequently and not touching the face [25]. The COVID-19 pandemic has raised a dilemma between economic stimulation and public health control [26]. As proposed in a dynamic modelling study, pandemic waves develop with the relaxation of public interventions [11, 20, 26]. Governments should be strict as well as considerate to all population segments when formulating policies. [21, 27]. In the absence of widespread vaccination, targeted approaches may be one of the best lines of epidemiological defence [21].

Moreover, in future suggestions that when a virus is widely circulating in a population and causing many infections, the likelihood of the virus mutating increases [28, 29]. The more opportunities a virus has to spread, the more it replicates [30, 31]. Priority should be given to vaccinating high-risk groups everywhere to global protection against new variants and minimize the risk of transmission [32]. As more people get vaccinated, virus circulation is expected to decrease, leading to fewer mutations [30].

This study has several limitations. Nationality and source population at risk factors were not directly estimated, and some gender and age information are missing. In addition, the calculation of mortality differences is at the limit of statistical significance. Therefore, our results must be taken with caution. However, we believe that the findings are relevant, as they might represent many similar centres in the Thailand area, and limited information is currently available on this issue.

## 5. Conclusion

The present study results show significant differences between five phases and three waves of the COVID-19 pandemic in Thailand, which may be attributable to a lack of strong social distancing policies and public health interventions. The most severe cases occurred among infants younger than years and adults over 60 years. The main risk sources were close contact with a previously confirmed patient and community risk. Early and timely interventions with strengthened social distancing policies should be implemented to effectively suppress and control the COVID-19 pandemic. In that context, border provinces have become a special focus of surveillance and screening activity. Generally, control measures have been tightened around the country, and people have been strongly admonished to wear masks, practice hand-washing hygiene, and be socially distant while outside the home.

We recommend acceleration of screening among workers so that they can enter the health service system as soon as possible and confirmed cases could be isolated. In addition, there should be a COVID-19 vaccination plan for individuals who are at risk of experiencing severe symptoms and the general population in outbreak areas to increase immunity in communities.

## Figures and Tables

**Table 1:** Cases and deaths across phases and waves during the COVID-19 pandemic.

Difference waves	Time period	Screening test *n* (%)	Confirmed case *n* (%)	Deaths *n* (%)
Phase I	January 2020	29,126 (0.0)	14 (0.0)	0
	February 2020	3,648,251 (32.0)	28 (0.0)	0

Phase II: first wave	March 2020	2,693,753 (24.0)	1,609 (1.0)	10 (1.0)
	April 2020	208,382 (2.0)	793 (0.4)	44 (4.5)
	May 2020	181,757 (2.0)	0	3 (0.3)

Phase III	June 2020	170,320 (1.0)	0	1
	July 2020	175,275 (2.0)	0	0
	August 2020	178,357 (2.0)	0	0
	September 2020	173,078 (2.0)	1 (0.0)	1
	October 2020	152,552 (1.0)	6 (0.0)	0
	November 2020	1,115,005 (10.0)	4 (0.0)	1

Phase IV: second wave	December 2020	293,928 (3.0)	2,415 (2.0)	1
	January 2021	518,255 (5.0)	11,454 (7.0)	16 (1.6)
	February 2021	378,673 (3.0)	6,858 (4.0)	6 (0.6)
	March 2021	405,822 (4.0)	2,585 (2.0)	11 (1.1)

Phase V: third wave	April–May 2021, continues	1,075,928 (9.0)	134,025 (84.0)	937 (90.9)
**Total**		**11,398,462 (100.0)**	**159,792 (100.0)**	**1,031 (100)**

**Table 2:** Comparison of sociodemographic characteristics during different phases during COVID-19 pandemic in Thailand.

Characteristics	Total of cases	Phases of the COVID-19 pandemic across 17 months					*p* value
		Phase I	Phase II first wave	Phase III	Phase IV second wave	Phase V third wave	
	*n* (%)	*n* (%)					
*Gender*							
Female	74,170 (48.5)	19 (45.2)	1,374 (45.2)	375 (40.0)	12,745 (58.0)	59,657 (47.0)	<0.001
Male	78,809 (51.5)	23 (54.8)	1,666 (54.8)	563 (60.0)	9,228 (42.0)	67,329 (51.5)	
**Total**	**152,979 (100)**	**42 (100)**	**3,040 (100)**	**938 (100)**	**21,973 (100)**	**126,986 (100)**	

*Age (years)*							
>1 (infants)	65 (0.0)	0	1 (0.0)	0	7 (0.0)	57 (0.0)	<0.001
1–3 (toddlers)	1,781 (1.2)	1 (2.4)	18 (0.6)	9 (1.0)	81 (0.4)	1,672 (1.3)	
4–5 (preschoolers)	977 (0.6)	0	12 (0.4)	2 (0.2)	48 (0.2)	915 (0.7)	
6–12 (middle childhood)	4,221 (2.8)	2 (4.8)	41 (1.3)	17 (1.8)	219 (1.0)	3,942 (3.1)	
13–15	1,921 (1.3)	0	29 (1.0)	10 (1.1)	155 (0.7)	1,727 (1.4)	
16–18	2,852 (1.9)	0	39 (1.3)	11 (1.2)	218 (1.0)	2,584 (2.0)	
19–24	22,264 (14.6)	2 (4.8)	370 (12.2)	148 (15.8)	3,511 (16.0)	18,233 (14.4)	
25–30	32,674 (21.4)	5 (11.9)	554 (18.2)	186 (19.8)	5,322 (24.2)	26,607 (21.0)	
31–35	21,520 (14.1)	10 (23.8)	369 (12.1)	135 (14.4)	4,000 (18.2)	17,006 (13.4)	
36–40	17,780 (11.6)	1 (2.4)	350 (11.5)	105 (11.2)	2,861 (13.0)	14,463 (11.4)	
41–50	23,482 (15.3)	4 (9.5)	557 (18.3)	171 (18.2)	3,141 (13.4)	19,609 (15.4)	
51–60	14,028 (9.2)	4 (9.5)	407 (13.4)	93 (9.9)	1,557 (7.1)	11,967 (9.4)	
>60	9,414 (6.2)	13 (31.0)	293 (9.6)	51 (5.4)	8,204 (3.9)	8,204 (6.5)	
**Total**	**152,979 (100)**	**42 (100)**	**3,040 (100)**	**938 (100)**	**21,973 (100)**	**126,986 (100)**	

*Nationality*							
Thai	118,391 (77.4)	17 (40.5)	2,734 (89.9)	689 (73.5)	7,922 (36.1)	107,029 (84.3)	<0.001
Foreigner	2,257 (1.5)	25 (59.5)	206 (6.8)	219 (23.3)	609 (2.8)	1,198 (0.9)	
Migrant worker	32,331 (21.1)	0	100 (3.3)	30 (3.2)	13,442 (61.2)	18,759 (14.8)	
**Total**	**152,979 (100)**	**42 (100)**	**3,040 (100)**	**938 (100)**	**21,973 (100)**	**126,986 (100)**	

*Source population at risk factors*							
Close contact with a previous confirmed patient	67214 (43.9)	32 (76.1)	1,599 (52.6)	276 (29.4)	2,773 (12.6)	62,534 (49.2)	<0.001
Health care worker	684 (0.4)	1 (2.4)	97 (3.2)	0	57 (0.3)	529 (0.4)	
Community risk	13,002 (8.5)	8 (19.0)	1,146 (37.7)	16 (1.7)	567 (2.6)	11,265 (8.9)	
Cluster community	39377 (25.7)	0	0	0	17,587 (80.0)	21,790 (17.2)	
Active and community surveillance	30,456 (19.9)	0	55 (1.8)	0	332 (1.5)	30,069 (23.7)	
State quarantine	2,246 (1.5)	1 (2.4)	143 (4.7)	646 (68.9)	657 (3.0)	799 (0.6)	
**Total**	**152,979 (100)**	**42 (100)**	**3,040 (100)**	**938 (100)**	**21,973 (100)**	**126,986 (100)**	

Statistical analysis was performed by chi-square test. *p* < 0.05 was considered to indicate statistical significance.

## Data Availability

The data used to support the findings of this study have been deposited in the Department of Disease Control in Thailand. The secondary analyses from announcement regarding the COVID-19 from the Department of Disease Control in Thailand data used to support the findings of this study are included within the article. The data used to support the findings of this study are restricted by the Ethics Committee of the Faculty of Medicine Vajira Hospital, Navamindradhiraj University, Bangkok, Thailand (Approval no. COE: 011/2021X) to protect patient privacy. The data are available from announcement regarding the COVID-19 from the Department of Disease Control in Thailand for researchers who meet the criteria for access to confidential data.
